# Chitosan–Hyaluronic Acid Nanoparticles for Active Targeting in Cancer Therapy

**DOI:** 10.3390/polym14163410

**Published:** 2022-08-20

**Authors:** Lisa Efriani Puluhulawa, I Made Joni, Khaled M. Elamin, Ahmed Fouad Abdelwahab Mohammed, Muchtaridi Muchtaridi, Nasrul Wathoni

**Affiliations:** 1 Department of Pharmaceutics and Pharmaceutical Technology, Faculty of Pharmacy, Universitas Padjadjaran, Sumedang 45363, Indonesia; 2 Department of Physics, Faculty of Mathematics and Natural Sciences, Universitas Padjadjaran, Sumedang 45363, Indonesia; 3 Functional Nano Powder University Center of Excellence (FiNder U CoE), Universitas Padjadjaran, Sumedang 45363, Indonesia; 4 Graduate School of Pharmaceutical Sciences, Kumamoto University, Kumamoto 862-0973, Japan; 5 Department of pharmaceutics, Faculty of pharmacy, Minia University, Minia 61519, Egypt; 6 Departement of Pharmaceutical Analysis and Medicinal Chemistry, Faculty of Pharmacy, Universitas Padjadjaran, Sumedang 45363, Indonesia

**Keywords:** cancer, chitosan, hyaluronic acid, nanoparticle, targeted delivery

## Abstract

Cancer is the most common cause of death worldwide; therefore, there is a need to discover novel treatment modalities to combat it. One of the cancer treatments is nanoparticle technology. Currently, nanoparticles have been modified to have desirable pharmacological effects by using chemical ligands that bind with their specific receptors on the surface of malignant cells. Chemical grafting of chitosan nanoparticles with hyaluronic acid as a targeted ligand can become an attractive alternative for active targeting. Hence, these nanoparticles can control drug release with pH- responsive stimuli, and high selectivity of hyaluronic acid to CD44 receptors makes these nanoparticles accumulate more inside cells that overexpress these receptors (cancer cells). In this context, we discuss the benefits and recent findings of developing and utilizing chitosan–hyaluronic acid nanoparticles against distinct forms of cancer malignancy. From here we know that chitosan–hyaluronic acid nanoparticles (CHA-Np) can produce a nanoparticle system with good characteristics, effectiveness, and a good active targeting on various types of cancer cells. Therefore, this system is a good candidate for targeted drug delivery for cancer therapy, anticipating that CHA-Np could be further developed for various cancer therapy applications.

## 1. Introduction

Cancer is the second leading cause of mortality globally after cardiovascular disease. Worldwide, there have been 19.3 million cancer diagnoses, with a death rate of about 10 million instances [1,2]. Cancer treatment options include surgery, radiation therapy, and chemotherapy. These treatments may cause serious side effects such as organ damage, spontaneous cell deaths, and a variety of other unexpected side effects, which may lead to non-compliant patients, resulting in their deaths [3,4,5]. Therefore, it takes effort to find effective treatments for cancer therapy. One of the promising cancer treatment strategies is the use of nanoparticle technologies [6]. A novel nanoparticle system that can be employed in the treatment of cancer is needed to increase drug effectiveness and to improve the drug release kinetics.

Among the various types of nanoparticles, polymeric nanoparticles, which are composed of polymers, can improve the physicochemical properties of drugs and deliver the active substances to their sites of action [7]. As a result of this technique, the active ingredient may be dissolved more easily, absorbed more readily, and accumulated in higher amounts in the tumor tissue site [8,9]. Compared with synthetic polymers, natural polymers offer better advantages in terms of absorption in the body, so the use of these polymers is encouraged [10].

Natural polymers are frequently used in pharmaceutical applications due to their biocompatibility compared with synthetic polymers [11]. As an example, the natural polysaccharide chitosan is commonly utilized in the synthesis of nanoparticles. Chitosan, an amphiphilic biopolymer produced by deacetylating chitin molecules, has mucoadhesive properties [12]. This polymer is the second most common polysaccharide polymer in nature after cellulose [10]. Amphiphilic polymer is an exceptionally valuable material for biotechnological, and pharmaceutical applications due to its biodegradability, biocompatibility, target specificity, and biostability properties [13]. The cationic charge of this polymer makes it special, because these cationic properties will make it functional to form interactions with other polymers through the formation of electrostatic complexes or multilayer structures [14,15]. Chitosan has been widely used as a polymer in nanoparticles as a drug carrier to produce nanoparticles with passive or active targeted delivery systems [16,17].

Active and passive targeting approaches are being employed to improve the effectiveness of various medications. Active targeting utilizes a ligand that has a specific affinity for a certain receptor. Nanoparticles are coated with a ligand to promote cellular absorption through receptor-mediated endocytosis, which increases the accumulation of medicines in cancer cells [18]. This method relies on the interaction between ligands on the surface of nanoparticles and cell surface receptors of cancer cells [19]. Hyaluronic acid (HA) is a polysaccharide ligand used chiefly for active targeting systems because of its high selectivity and affinity to CD44 receptors highly expressed in different tumor cells [18,20].

**Figure 1:**
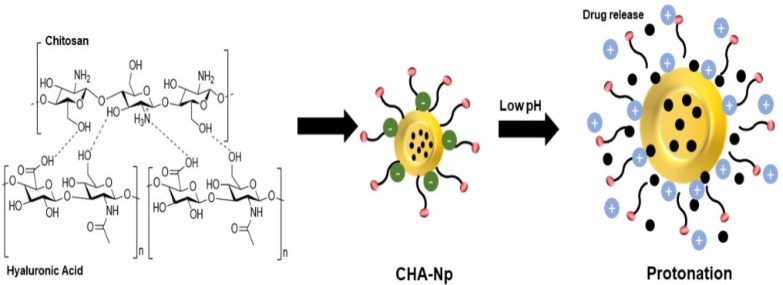
Formation of an electrostatic complex between the negative charge of hyaluronic acid and the positive charge of chitosan and the pH-responsive behavior.

Chitosan–hyaluronic acid nanoparticles (CHA-Np) are nanoparticles composed of chitosan and hyaluronic acid ligands, both of which are natural polysaccharides [21,22,23,24]. Since both polymers have opposite charges, formulation of nanoparticles is simpler, requiring modifications in electrostatic interaction between the positive charge of chitosan and the negative charges of hyaluronic acid (Figure 1) [25,26], compared with other nanoparticle ligand systems (such as folic acid, trastuzumab, estrogen, etc.) requiring extensive chemical modifications.

CHA-Np offers a controlled release of drugs with a pH-stimuli responsive, owing to the presence of the amide group in chitosan, which will be protonated at low pH, resulting in improved drug release [27]. It is exploited by researchers to deliver cancer therapeutics to deliver drugs to cancer cells, which are known to have a lower pH than normal cells. CHA-Np also offers active targeting due to the presence of hyaluronic acid ligands, which makes these nanoparticles widely used. The high selectivity of hyaluronic acid and CD44 receptors results in accumulation of these particles inside the tumor cells expressing CD44 [22].

Currently, CHA-Np has been widely prepared using a mixture of polymers or by other development approaches. In addition, CHA-Np is widely utilized as a selective carrier for many solid tumor types, such as brain tumors, breast cancer, lung cancer, liver cancer, and colon cancer. However, the expression level of this receptor varies from one cell type to the other. Because of this, published evidence was collected and evaluated to explain the importance and effectiveness of CHA-Np in various types of cancer cells in the expectation that this study might be utilized as a reference and consideration for further development.

## 2. Methodology

**Figure 2:**
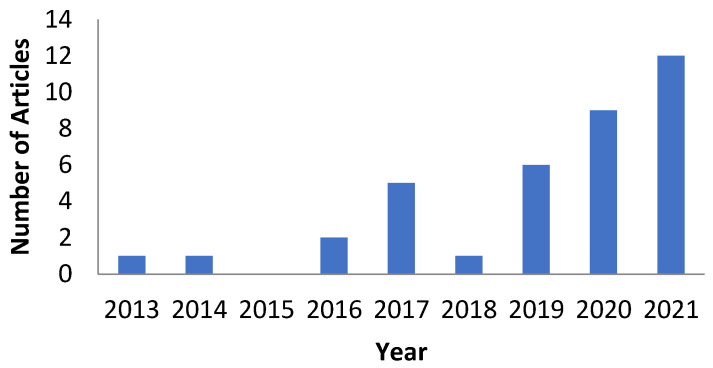
Number of total articles used by year.

**Figure 3:**
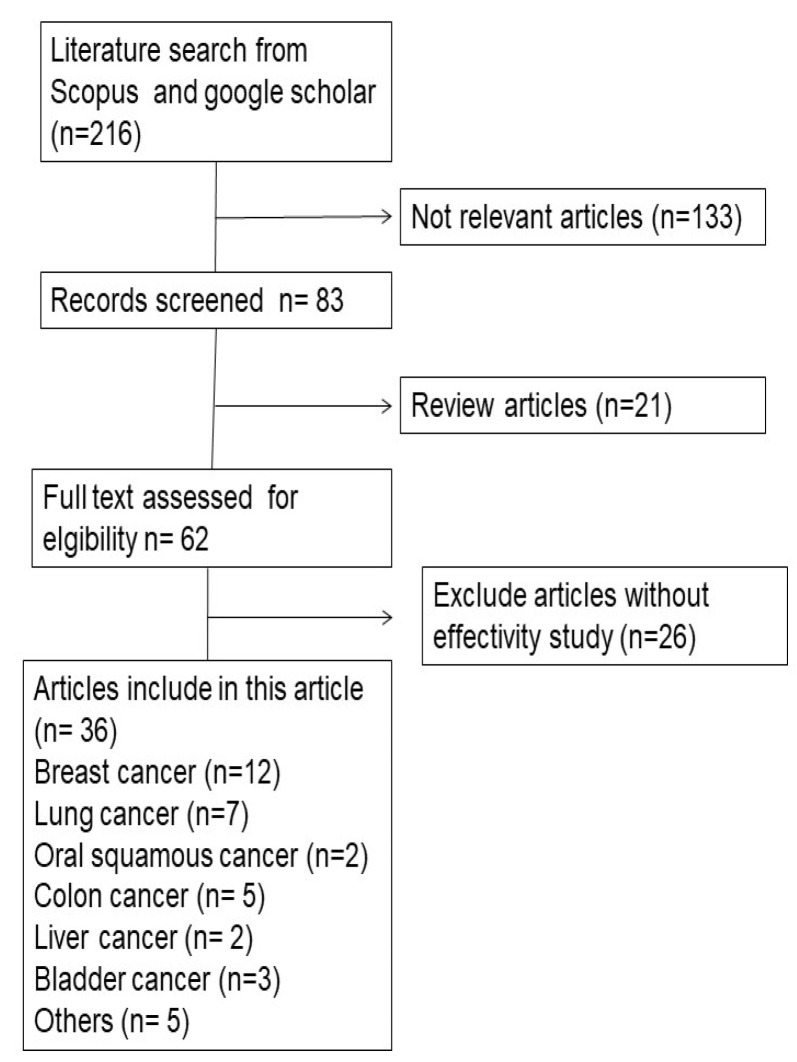
Flowchart of the methodology.

The preparation of this review was based on the results of gathering and review articles from several sources namely Scopus, Science Direct, and Google Scholar using the keywords “Chitosan Hyaluronic Acid Nanoparticle”, “Chitosan Hyaluronic Acid Nanoparticle for Cancer Therapy”, “Chitosan Hyaluronic Acid Nanoparticle for Breast Cancer”, “Chitosan Hyaluronic Acid Nanoparticle for Lung Cancer”, and so on. The inclusion criterion was journals with in vitro and in vivo testing, while the exclusion criterion was article reviews. In the first search results, 216 articles were found. After reading the titles and abstracts, 36 articles were selected to be reviewed comprehensively and included in this review (Figure 2). The flowchart of the methodology is shown in Figure 3. The development of the CHA-Np formulation (according to the criteria) during 2019–2021 continues to increase, with the highest number of development articles in 2021 (12 articles); in this year, many types of delivery systems have been developed with active targeting uses of CHA-Np.

## 3. Polymeric Nanoparticles

Polymeric nanoparticles are composed of polymers that help protect and deliver active substances [28,29]. Depending on the source of polymers, the type of polymer in the preparation of nanoparticles consists of two types, namely synthetic and natural polymers. Synthetic polymers are polymers derived from modifications and chemical reactions. In contrast, natural polymers are polymers derived from natural materials such as animals and plants [30,31]. When choosing polymers for oral or parenteral formulations of nanoparticles, they should be biodegradable as well as biocompatible and non-immunogenic.

Chitosan is one of the natural polymers from the polysaccharide group, which are biodegradable, biocompatible, and non-immunogenic, originating from the chitin deacetylation extracted from shrimp, crab, and freshwater lobster [10]. Chitosan has been widely used as a polymer for nanoparticle formulations [32,33,34]. Chitosan is also able to deliver drugs in nanoparticle systems [35,36]. Chitosan nanoparticles offer controlled release of drugs through the process of degradation and corrosion of chitosan. Hydrophobic alterations of chitosan and nanoparticle arrangement by self-assembly in fluid arrangement adsorb hydrophobic drugs with high adequacy and discharge them in a sustained release [37]. This polymer is reported to have an amphiphilic chain that can interact with cell membranes and also has an adsorption effect on the cell surface. As a result, it has the potential to be an excellent drug carrier [13,38]. It was reported that chitosan nanoparticles had a long circulation time (prevent adhesion) and selectivity in cancer cells (pH-sensitive) [39].

Chitosan nanoparticles can be created using various procedures, including precipitation, ionic crosslinking, covalent crosslinking, polymerization, and other techniques [38]. Chitosan is generally treated with a crosslinking agent such as tripolyphosphate to achieve the desired particle size, with the greater crosslinking resulting in a more solid particle. As a consequence, it may aid in the drug’s entrapment [35,36]. Because most polysaccharides have a neutral or negative charge in an acidic environment, this polymer is a cationic polymer, which distinguishes chitosan. These cationic characteristics will allow it to create electrostatic complexes or multilayer structures with other polymers and active ingredients [14,15].

## 4. Hyaluronic Acid Nanoparticles as an Active Targeting Drug Delivery System

The targeted delivery system consists of two types of delivery: passive targeted drug delivery and active targeted drug delivery. The passive targeted drug delivery system is based on increasing permeability and retention effects. In contrast, the active–passive targeted drug delivery system depends on ligand affiliation to receptors [40]. The active–passive targeted drug delivery system is one of the ligand-assisted drug targeting strategies, wherein ligands bind to receptors. Ligands will be conjugated on the surface of nanoparticles, resulting in increased cellular absorption via endocytosis-mediated receptors, increasing drug accumulation in cancer cells. The basis for this process is the interaction between ligands conjugated on the surface of nanoparticles and cell surface receptors or antigens on the surface of cancer cells [20]. One of the ligands that is widely used to target drugs is hyaluronic acid [41,42,43,44].

Hyaluronic acid is a biodegradable, biocompatible, and non-toxic natural polysaccharide widely used in nanoparticle formulation. Hyaluronic acid is a non-sulfated glycosaminoglycan that is highly hydrophilic and composed of disaccharide-glucuronic acid and N-acetyl-D-glucosamine units. It is produced naturally in the body. These polysaccharides can activate cellular communication by attaching to CD44 receptors, which are also found throughout the body, particularly in cancer cells [45,46]. In this way, hyaluronic acid acts as a ligand in a targeted delivery system by binding to CD44 receptors [45,46]. The interaction between CD44 receptors and hyaluronic acid ligands regulates BCL-2 release in the cell, preventing apoptosis, increasing cell proliferation, and improving cellular motility. The ligand of CD44 receptors is utilized to aggressively target medicines through this interaction, notably in cancer cells [47].

## 5. Chitosan–Hyaluronic Acid Nanoparticles for Cancer

Hyaluronic acid chitosan nanoparticles are nanoparticles composed of chitosan polymers with hyaluronic acid ligands aimed at targeting drugs actively to receptors that are widely expressed in cancer cells [48,49,50]. The use of these two natural polysaccharides makes these nanoparticles safer and easy to produce [30]. In its formulation, CHA-Np systems have been developed, starting from the addition of new polymers or developing the formulation system as shown in the following Table 1:

### 5.1. Breast Cancer

Breast cancer is the most common cancer in women, as well as the leading cause of death [2]. The CD44 receptor has been found to be overexpressed in some types of breast cancer [85]. Therefore, many therapeutic developments have been carried out against this cancer using hyaluronic acid ligands [54,70,71]. Deng et al. (2014) used the ionic gelation technique to successfully encapsulate miR-34a and doxorubicin with chitosan polymers and hyaluronic acid ligands in CHA-Np, having a particle size of around 171–214 nm. These findings suggest that these nanoparticles could increase doxorubicin’s anticancer impact by inhibiting the production of Bcl-2’s non-pump-resistant and anti-apoptotic proto-oncogene. Furthermore, miR-34a also inhibited breast cancer cell migration on the MDA-MB-231 cell line by targeting Notch-1 signaling [51].

Another study employed a hyaluronic acid and chitosan system to encapsulate cisplatin and doxorubicin using the chelation reaction technique, and Schiff’s base was evaluated on the MCF-7 cell line. The results revealed that this approach enhanced cisplatin and doxorubicin absorption while also increasing cytotoxic activity when the particle size was 160 nm [52]. Ravari et al. (2016) conducted another investigation in which they used the polyelectrolyte complex technique to conjugate chitosan–hyaluronic acid with docetaxel. The nanoparticles had a particle size of around 170–210 nm and IC50 values of 45.34 µM and 354.25 µM in cell lines 4 T1 and MCF-7, respectively, while docetaxel alone had IC50 values of 233.8 µM and 625.9 µM. A reduction in the IC50 number shows that the drug’s efficacy has improved [53].

Rezaei et al. (2020) also succeeded in formulating CHA-Np with lipoic acid to encapsulate methyltestosterone through the ionic gelation method. The results showed that particle size was 280 nm, and were evaluated against the MCF-7 breast cancer cell line. The results showed a higher absorption of nanoparticles in cells than the single active substance. Not only this, but a slow-release profile also resulted from this system [54]. The formulation of letrozole using the CHA-Np technology has also been evaluated. This formulation was evaluated in vivo and included PLGA as an extra polymer. As a result, this nanoparticle system resulted in nanoparticles with size of 464 nm, a regulated release, and which were tolerated by lab mice [55]. The ionic gelation technique was also utilized to encapsulate methotrexate using the CHA-Np technology. This system showed a small particle size (190–300 nm) and when compared with free methotrexate, this formulation demonstrated an increase in anticancer activity when tested in the MCF-7 cell line [56].

The synthesis of CHA-Np with the addition of oleic acid to encapsulate bismuth carried out by the sol-gel method showed the production of small particles (10–20 nm) that were able to inhibit the growth of cancer cells in the MCF-7 cell line, and the results of in vivo testing showed that the presence of chitosan was able to increase the half-life and prevent adhesion on nanoparticles [48]. Another synthesis was carried out using chitosan polymer and Di (ethylene glycol) methyl ether methacrylate with the addition of the hyaluronic acid ligand, tested against the MDA-MB-231 cell line. The obtained results showed that these nanoparticles with a particle size 190 nm could eradicate cancer cells by decreasing cell viability and increasing cancer cell apoptosis. In vivo testing results showed that the distribution of these nanoparticles is at the cancer site, while the free drug is distributed more widely and is excreted more quickly from the body [57]. Another study focused on the production of CHA-Np, which was used to entrap doxorubicin, combined with tripolyphosphate via a complicated electrostatic process before even being reacted with chitosan. Hyaluronic acid served as a ligand at the same time. These nanoparticles had a 220–280 nm particle size and were found to induce apoptosis, and in vivo tests on SEC (Solid-form of Ehrlich Carcinoma) demonstrated good antitumor efficacy [58,65].

Other CHA-Np experiments were also carried out with the addition of PEG (poly (ethylene glycol)) to entrap 3-fluoro-4-carboxy phenylboronic acid. The results showed that particle size of this nanoparticle was 200–330 nm and exhibited obvious anticancer activities in the MCF-7 and MDA-MB-231 cell lines with IC50 values of 0.14 and 0.05 µg/mL, respectively [60], comprising a three-layer nanoparticle system using PEG, hyaluronic acid, and chitosan. The first layer is paramagnetic iron oxide, the middle one contains chitosan with hexadecanol, and the outer layer is hyaluronic acid and PEG. The results of this study indicated that this layered nanoparticle system with a size of 220 nm can deliver the drug directly to the cancer site and produce an excellent therapeutic effect on MDA-MB-231 cancer cells [60,67]. A CHA-Np system was utilized to entrap liquid crystalline nanoparticles of tamoxifen and resveratrol, which were then encapsulated with chitosan–hyaluronic acid again. These nanoparticles had a particle size of 217 nm and reduced cell growth in the MCF-7 cancer cell line in vitro but not in normal cells. Increasing cellular absorption owing to hyaluronic acid’s presence was another promising finding of this investigation [61]. In comparison with the conventional anticancer drugs without the ligand, the addition of hyaluronic acid permitted the drug to act effectively at the tumor site.

### 5.2. Lung Cancer

Lung cancer has the second greatest incidence after breast cancer [2]. Because most lung cancer cells highly express CD44 receptors, hyaluronic acid can be used as a ligand to target these cells [86,87,88,89]. Many investigations have been conducted on the production of CHA-Np actively targeting pharmaceuticals. Zhang et al. (2019) created siRNA nanoparticles using chitosan/hyaluronic acid and cyanine-labeled nanoparticles. A549 comprises lung cancer cells that overexpress the CD44 receptor. These nanoparticles had a particle size of 127 nm and could suppress the cell growth by down-regulating the BCL2 target gene, according to the study’s findings. However, in vivo studies reveal that nanoparticles are more effective than a single active ingredient at reaching the target site [62].

Another study used the ionic gelation method for preparing CHA-Np with the active component 5-fluorouracil. These nanoparticles had a particle size of 119 nm and significant inhibitory potential for cancer development and could enhance apoptosis compared with a single active ingredient in these nanoparticles. ROS (reactive oxygen species) produced by the nanoparticles might potentially cause mitochondrial cell death [63]. CHA-Np was also produced to encapsulate doxorubicin and celecoxib, albeit chitosan glycol was used. A pH-sensitive crosslinking method is used in the manufacturing process. The findings indicated that the two active chemicals had a synergistic impact. In vivo results showed that, when compared with a single active chemical, the nanoparticles decreased the tumor growth of lung cancer cell types, as well as inflammation and metastasis [64].

Other nanoparticles were created by the trapping of raloxifene and chitosan polymers, as well as a ligand for active targeting, mainly hyaluronic acid. The nanoparticles with a size of 142 nm exhibited a strong cytotoxic effect on the A549 cells, reducing cell viability through decreased glucose absorption, which diminished bioenergy, and activating apoptosis through increased nitric oxide levels [65]. Ionic gelation was used to generate CHA-Np that shields and protects peptides from oxidative damage and degradation. According to the study’s findings, drug excretion was faster at acidic pH than at physiological pH. It was also demonstrated that nanoparticles with a particle size of 140–240 nm had considerable anticancer action, and that a drug had a greater effect on the cytotoxicity test than nanoparticles. To the best of the authors’ knowledge, the CHA-Np will not damage normal human cells (selectively) [66].

Another study used the LBL (layer by layer) technique to create CHA-Np with the addition of a PCL polymer to entrap naringenin (self-assembling). When modifying the two polyanion layers, the polymeric polycationic layer serves as a connection. The result showed that nanoparticles with a particle size of 251 nm exhibited a significantly higher cellular absorption than a single active chemical and were also efficient in preventing the development of cancer cells in experimental animals [67]. The findings of gambogic entrapment utilizing a CHA-Np system (212 nm) revealed great cellular uptake, fast drug release, and enhanced apoptosis in A549 lung cancer cells when compared with the active component without nanoparticle formulation [68]. These results indicate that the use of hyaluronic acid can increase cellular absorption compared with nanoparticles without hyaluronic acid.

### 5.3. Liver Cancer

Liver cancer is a type of cancer that targets human liver cells, and it ranks fourth in terms of global prevalence [2]. The CD44 receptor, which binds to hyaluronic acid, is also expressed in liver cancer [90,91,92]. This is supported by prior studies on active targeting in these cancers. In one of their studies, Li et al. (2013) utilized ionic gelation to create CHA-Np to entrap paclitaxel. This study’s findings suggested that nanoparticles with a particle size of 100 nm show pH-dependent release. Through receptor-mediated endocytosis, the nanoparticles exhibited more cellular absorption than the free active substance against the HepG2 cell line. Active material accumulated significantly at the tumor site due to the presence of actively targeted ligands [69].

In another study, Sato et al. (2017) used chitosan–hyaluronic acid to produce gene (pDNA) nanoparticles. This study demonstrated that these nanoparticles may successfully retain microencapsulated pDNA ternary complexes, transfecting cells to form nanoparticles with particle sizes of 203–390 nm. The results of in vivo testing on mice with lyophilized and dehydrated pDNA ternary complex cancer indicated that tumor development was successfully impaired [70].

### 5.4. Oral Cavity Squamous Cancer

Oral cavity cancer or Squamous Cell Carcinoma (SCC) of the oral cavity is a malignant tumor that attacks the oral cavity with a high incidence and recurrence rate in the world. This type of cancer has also been reported to overexpress the CD44 receptor, which is the receptor for hyaluronic acid [93,94]. Therefore, several studies regarding the active targeting of drugs at these receptors have been carried out.

One study was conducted on the production of CHA-Np for this oral cavity cancer, specifically for squamous carcinoma, both squamous mouth and throat. The findings of Huang et al. (2021), who generated nanoparticles using chitosan and PCL polymers and hyaluronic acid ligands and evaluated them in vitro and in vivo, revealed that nanoparticles were absorbed at the cancer cell site EC109 via an endocytosis process via the CD44 receptor. Furthermore, these nanoparticles with particle sizes around 266–402 nm preferentially target cancer cells while not affecting NIH3T3, a normal fibroblast cell. The findings of in vivo tests demonstrated that these nanoparticles are safe to provide to experimental animals and can deliver drugs to tumor tissue via oral administration [76].

The ionic gelation approach was also used to effectively synthesize catechol nanoparticles (pyrocatechol or 1,2-dihydroxybenzene) using chitosan polymer and hyaluronic acid ligand. These nanoparticles (160 nm) are targeted at HN22, a squamous cancer cell (SCC) of the oral cavity. The obtained results revealed nanoparticles with a delayed release. However, the potential to inhibit the growth of cancer cells with a low IC_50_ value might enhance apoptosis and the accumulation of the active drug at the cancer site as compared with a single active substance [77]. The findings of the previous studies indicate that the CD44 receptor is expressed in this malignancy, and the use of hyaluronic acid can promote the absorption of nanoparticles in cells.

### 5.5. Colon Cancer

Colon cancer is the third most prevalent cancer after breast cancer and lung cancer. The CD44 receptor has also been overexpressed in colon cancer [95,96,97]. Therefore, several studies on targeted delivery in cancer have been carried out using hyaluronic acid ligands. The fabrication of CHA-Np to encapsulate mRNA has been successfully tested on HCT116, human colon cancer cells. The results revealed that the CHA-Np system transported mRNA to the tumor site and formed a stable system. Furthermore, this nanoparticle system with particle size of 200–300 nm performed best in an acidic environment (pH 6.4), which is the same as the cancer cells’ milieu, which has a lower pH than normal [51,71].

Sharifi et al. (2021) created nanoparticles using Methoxy poly (ethylene glycol)/chitosan co-polymers and hyaluronic acid ligand to entrap the 7-ethyl-10-hydroxycamptothecin, which was evaluated against HCT-116 colon cancer. To conjugate the active ingredient, ionic gelation and physisorption were utilized in this synthesis and produced 227 nm nanoparticles. In vitro, these nanoparticles were able to suppress cancer cell growth and enhance absorption by 1.6 times in the Caco-2 cell line. This is considered to be related to the presence of ligands, which are utilized to enhance nanoparticle absorption [72].

In addition, CHA-Np was utilized to entrap super magnetic iron oxide nanoparticles and TAT peptides for siRNA delivery against STAT3 and HIF-1 α. The nanoparticles (118 nm) inhibited proliferation, migration, and increased apoptosis in the CT26 cell line according to the findings of this study [73]. Other nanoparticles were used to deliver anti-IL-6 and BV6 utilizing chitosan–hyaluronic acid-PEG. The results indicated that this nanoparticle size was 100 nm and this combination may enhance apoptosis while decreasing cell migration, proliferation, and colony formation in the cell lines 4T1 and CT26. When these nanoparticles were delivered to the experimental animals, they likewise exhibited an increase in survival time [74]. In addition, carboxylate graphene oxide and trimethyl chitosan coupled with hyaluronic acid-based ligands were used to produce CHA-Np to entrap HIF-1α siRNA and dinaciclib (95 nm). They were able to inhibit CDK/HIF-1α in cancer cells, and decreased proliferation, migration, angiogenesis, and colony formation in the CT26 colon cancer cells according to this report [75].

### 5.6. Bladder Cancer

Bladder cancer has a significant mortality rate, and it commonly develops despite administration of appropriate treatment [98]. Males are more likely than females to have this cancer [99]. Several researchers revealed that this cancer likewise expresses the CD44 receptor, enabling therapeutic targeting with hyaluronic acid in such tumors [100,101,102]. Nanoparticles were created by Liang et al. (2021) to target highly expressed CD44 T24 bladder cancer cells, enabling the usage of hyaluronic acid to be executed when linked with CD44–hyaluronic acid ligands; this nanoparticle system loaded with siRNA and delivered it to cancer sites while also possessing preferential accumulation in vivo for the Bcl-2 oncogene to target bladder cancer [23].

Another study from Salehi et al. (2021) synthesized CHA-Np with HIV-1 derivative TAT peptide (Trans-Activator of Transcription) for the treatment of bladder cancer, also carried out by delivering CD73 siRNA and doxorubicin with a particle size of 118 nm. The results showed that these nanoparticles could increase cell death and inhibited cell proliferation and absorption, while the in vivo test results showed an increase in the survival of experimental animals, reduced tumor growth, and enhanced the immune response against cancer cells [78]. Several cancer medicines can target HIF-1-α (hypoxia-inducible factor-1-α) because they are essential in cancer cell growth. For bladder cancer, nanoparticles containing super magnetic iron oxide, chitosan, and hyaluronic acid adsorb HIF-1 and prostaglandin E2. The particle size of these nanoparticles was 130 nm. The interaction of hyaluronic acid with the CD44 receptor enhanced nanoparticle accumulation in cancer cells and inhibited cell proliferation, migration, invasion, angiogenesis, and colony formation in T24 cells, suggesting that these nanoparticles could actively deliver drugs to the cancer site [79].

Utilizing confocal and film imaging, Akentieva et al. (2020) found out that doxorubicin nanoparticles were localized around cells and in the nucleus after using the ionic gelation method to synthesize the drug on a CHA-Np system. In addition, this method could increase the effectiveness of the active ingredient [84].

### 5.7. Others

In addition to the types of cancer mentioned above, researchers have used CHA-Np for active targeting to other cancer cells, such as Gao et al. (2017), who formulated CHA-Np with the addition of poly (lactic-co-glycolic acid) (PLGA) co-polymers to entrap irinotecan and 5-fluorouracil. Researchers found that the nanoparticle (153 nm) formulation could increase the anticancer activity of two medicinal compounds in mice inoculated with stomach cancer. Hyaluronic acid has also been shown to enhance the direct delivery of nanoparticles to cancer sites [80]. CHA-Np was also used to encapsulate Nitric Oxide in the treatment of prostate cancer. This system had a size of 142–180 nm and has a relationship between nitric oxide concentration and its anticancer action, indicating that more nitric oxide has a much more potent anticancer effect [81].

In addition, graphene oxide CHA-Np was created. In this work, chitosan was first transformed into carboxymethyl chitosan. According to the study, this system’s drug release was higher in a cancer pH environment than in a normal pH environment. According to the results of the cellular absorption tests, these nanoparticles targeted cells with high expression of the CD44 receptor and inhibited their proliferation [82]. In another study for brain cancer therapy, curcuminoids were entrapped with chitosan polymer and hyaluronic acid ligands with PEG. The findings revealed that these nanoparticles (particle size 210–240 nm) could cross the BBB (Blood–Brain Barrier) and remained in the brain for 72 h, increasing the drug’s efficiency when formulated as nanoparticles [83].

## 6. Author Perspective

To deliver therapeutics specifically to cancer areas, CHA-Np utilizes chitosan to entrap active substances [66]. They are therefore safe to use [14,15]. The ionic gelation technique is one of the methods used to prepare CHA-Np. This approach is widely utilized since chitosan is a positively charged polysaccharide (cation) and hyaluronic acid is negatively charged (anion) [15]. This CHA-Np formulation will produce suitable particle size and bonding at a pH ranging from 5–6. Therefore, this pH range is the only one where these two polymers can completely ionize and form strong electrostatic interactions [41]. Nanoparticle formulations for doxorubicin entrapment [84], paclitaxel entrapment [69], peptide entrapment [66], curcuminoids entrapment [101], and other nanoparticle formulations require ionic gelation processes.

At present, we have found that the nanoparticle system of chitosan and hyaluronic acid can produce molecules with desirable characteristics. The particle size of CHA-Np is <100–400 nm depending on the amount and type of polymer used, where the smaller the particle size, the faster solubility and the higher bioavailability of the drug. This small particle size is also suitable for improving permeability and retention (EPR), thus improving drug bioavailability in the body [103]. Generally, the optimal particle size for cancer tissue-targeted drug delivery systems is a size of 100–200 nm with maximum entrapment efficiency [104]. Due to hyaluronic acid’s negative charge, CHA-Np has a lower zeta potential than those without hyaluronic acid [105]. Higher negative zeta potential means a higher diffusion coefficient and nanoparticles will penetrate cells faster compared with positively charged nanoparticles [106]. The shape of nanoparticles is also generally spherical, which is advantageous in the internalization of the nanoparticles due to their symmetrical shape, so it does not require special attention to the contact angle formed between the nanoparticles and the cell surface [107]. CHA-Np may also extend the half-life of drugs due to the encapsulation process since swelling of the matrix occurs earlier and drug release is more steady [48,79,108], showing a pH–stimulus responsiveness, where the change in pH can affect the release of the active substance from the nanoparticle system. This is evidenced by the results of drug release testing from CHA-Np, which is higher at low pH than at the physiological pH of the body. This is because at low pH, the amine group of chitosan swells due to protonation, thus allowing the active substance to escape from the nanoparticles (Figure 1) [27,51].

When combined with other polymers, chitosan can also easily interact through the formation of electrostatic complexes or multilayer structures [14,15]. Several polymers have been used to combine CHA-Np, such as the layer-by-layer nanoparticle formulation used to entrap tamoxifen and resveratrol. These two active ingredients were combined using a liquid crystalline nanoparticle system (Liquid Crystalline Nanoparticles), which is then enveloped in chitosan polymer and hyaluronic acid ligand. The goal of this study was to improve the drug’s efficacy against cancer cells. While the drug release was increased in the liquid crystalline nanoparticle system, it was slower in CHA-Np due to the excellent entrapment of chitosan and hyaluronic acid [60,109]. Other polymers, such as PCL, have been used in the formation of CHA-Np. Chitosan was also used to create multilayer nanoparticles by connecting hyaluronic acid and PCL. The particle size increased with the addition of this polymer, and the drug release was prolonged because the breakdown medium takes a long time to infiltrate the system [67].

**Figure 4:**
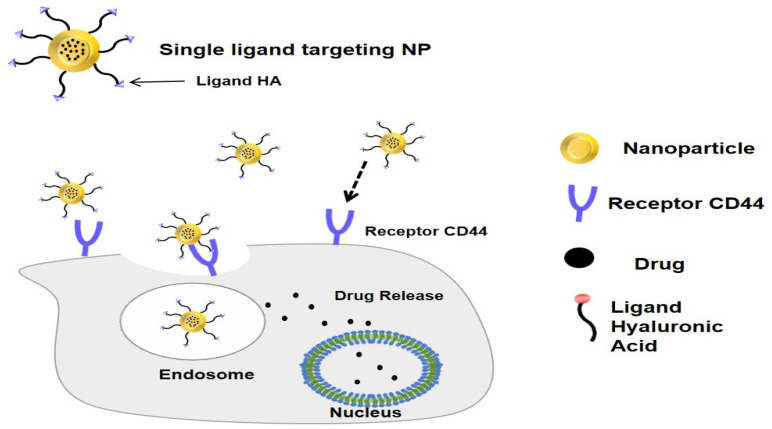
Internalization of CHA-Np in cancer cells by the CD44 receptor.

The efficiency of CHA-Np has been demonstrated in many cancer cell lines (in vitro), with an increase in apoptosis-inducing proteins, decreased proliferation, mutation, and angiogenesis, as well as the inhibition of cell viability [63,73]. Whereas in vivo, which was tested mostly on mice that had been inoculated with cancer cells, these nanoparticles were able to reduce tumor volume and size, the conjugated hyaluronic acid was able to deliver nanoparticles to cancer sites more than in other normal tissues [76,79]. Improved efficacy is attributed to the CHA-Np combination’s increased internalization (Figure 4) [101], resulting in improved endocytosis process with the addition of a ligand, hyaluronic acid, specifically targeting the CD44 receptor. Moreover, the adverse effects will be minimized and drug efficacy will enhanced due to nanoparticle targeting [53,54].

**Figure 5:**
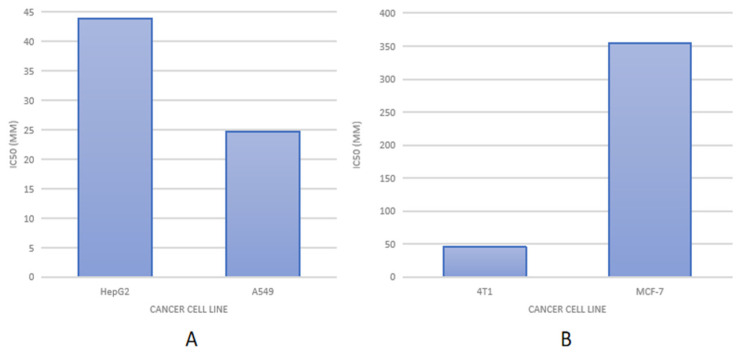
IC50 of CHA-Np in various cancer cells. (**A**) Raloxifen loaded in CHA-Np, (**B**) Methyltestosterone loaded in CHA-Np.

There has been extensive research into the use of hyaluronic acid as a drug delivery system for treating several types of cancers. Studies on breast cancer therapy are the most intensive, followed by lung cancer and bladder cancer. Therefore, CHA-Np therapy can enhance the drug’s efficacy because of the overexpression of the CD44 receptor in these cancer types. It has been shown that the expression of CD44 receptors varies between different types of malignancies (Figure 5).

The differences in the expressions of the hyaluronic acid receptor (CD44) in different cells affect the amount of drug accumulation in cancer cells. The less receptor expression, the less CHA-Np can be internalized, so its effectiveness and inhibition of cancer growth will be significantly lower. As in Figure 5, the IC50 of HepG2 (IC50 43.80 µM) is greater than in lung cancer A549 (IC50 24.60 µM) [65], and IC50 in the MCF7 (IC50 354.25 µM) cell line is greater than in the 4T1 (IC50 45.34 µM) cell line [53]. This shows that the expression of the CD44 receptor in the HepG2 cell line is lower than the A549 cell line. Moreover, the CD44 receptor was expressed more in triple-negative breast cancer 4T1 than in MCF7 cell lines. As such, before creating this system, it is crucial to first consider the expression level of a cell’s receptors in the target cell. This will help create an effective drug delivery system [63]. Therefore, the use of CHA-Np for targeting cancer showed a significant increase in tumor accumulation, cellular absorption, and eventually a stronger tumor cytotoxic effect. Consequently, this system represents a promising candidate in targeted drug delivery for cancer therapy.

This is due to differences in the expression of the hyaluronic acid receptor (CD44) in different cells.

## 7. Conclusions

Hyaluronic acid–chitosan nanoparticles are biodegradable and non-toxic nanoparticle systems that can easily form through electrostatic interaction between these two polysaccharides due to the opposite charges they carry. To achieve excellent drug absorption, either single CHA-Np or modified nanoparticles containing additional polymers maybe employed. They can improve drug absorption in cancer cells by attaching directly to the CD44 receptor, resulting in enhanced therapeutic efficacy in cancer cells, including breast cancer, lung cancer, bladder cancer, colon cancer, oral squamous cavity (SCC) cancer, cervical cancer, liver cancer, and others. However, the expression of the CD44 receptor varies depending on the type of cancer. For instance, its expression in hepG2 and Huh-7 is significantly lower than in A549 cell lines. Therefore, researchers need to investigate the expression of this receptor in target cells before designing an effective targeted drug delivery system using hyaluronic acid ligands in order to maximize the effectiveness of the system.

## Figures and Tables

**Table 1:** Chitosan–hyaluronic acid nanoparticles for cancer therapy.

No	Cancer Types	NDDS	Particle Size (nm)	Zeta Potential (mV)	Cell Line	Testing	Activities *In Vitro*			Activities *In Vivo*		Ref.
							ITG	Ap	CU	TV	S	
1.	Breast cancer	miR-34a, Dox, C, HA	214	−33	MDA-MB-231	In vitro	√	√	-	-	-	[51]
2.		Cisplatin, Dox, C, HA	160	−28	MCF-7	In vitro	√	-	√	-	-	[52]
3.		DTX, C, HA	170–210	18–24	MCF-7 and 4T1	In vitro	√	-	√	-	-	[53]
4.		Methyltestosterone, lipoic acid, C, HA	280	19	MCF-7	In vitro	√	√	√	-	-	[54]
5.		Letrozole, C, HA, PLGA	464	−10.5	MCF-7	In vitro and in vivo	√	√	√	√	-	[55]
6.		Methotrexate, C, HA	190–300	−20–(−30)	MCF-7	In vitro	√	-	-	-	-	[56]
7.		Bismuth, oleic acid, C, HA	10–20	−30.9	MCF-7	In vitro and in vivo	√	-	-	√	-	[48]
8.		Pacitaxel, di(ethylene glycol) methyl ether methacrylate, C, HA	190	-	MDA-MB-231	In vitro and in vivo	√	√	√	√	√	[57]
9.		Dox, TPP, C, HA	220–280	-	MCF-7	In vitro and in vivo	√	√	-	√	-	[58]
10.		3-fluoro-4-carboxyphenylboronic acid, PEG, C, HA	200–330	−10.8	MCF-7 and MDA-MB-231	In vitro	√	-	√	-	-	[59]
11.		PEG, C, HA, hexadecano, Gamboic acid	220	45	MDA-MB-231	In vitro	√	-	√	-	-	[60]
12.		Tamoxifen, resveratrol, poloxamer, C, HA.	217	17.5	MCF-7	In vitro and in vivo	√	√	-	√	√	[61]
13.	Lung cancer	Cyaine3 labeled siRNA, C, HA	127	31	A549	In vitro and in vivo	√	-	√	√	√	[62]
14.		5-Fluorouracil, C, HA	119	15.6	A549	In vitro	√	√	√	-	-	[63]
15.		Dox, Celocoxib, C, HA	150	−25	A549	In vitro and in vivo	√	√	√	√	-	[64]
16.		Raloxifen, C, HA	142	−15	A549	In vitro	√	√	√	-	-	[65]
17.		Peptide CM11, C, HA	140–240	51	A549	In vitro	√	√	-	-	-	[66]
18.		Naringenin, PCL, C, HA	251	−19.5	A549	In vitro and in vivo	√	-	√	√	-	[67]
19.		Gamboic acid, C, HA	212	−23	A549	In vitro and in vivo	√	√	√	√	√	[68]
20.	Liver cancer	Paclitaxel, C, HA	100	−11	HepG2	In vitro	√	-	√	-	-	[69]
21.		pDNA, C, HA	203–390	−37	Huh7	In vitro	√	-	-	-	-	[70]
22.	Colon cancer	mRNA, C, HA	265–350	−40	HCT-116	In vitro	√	-	-	-	-	[71]
23.		7-ethyl-10-hydroxycamptothecin, PEG, C, HA	227	-	HCT-116	In vitro and in vivo	√	-	√	√	-	[72]
24.		siRNA, TAT peptide, C, HA	118	20	CT26	In vitro	√	√	-	-	-	[73]
25.		anti-IL-6, BV6, PEG, C, HA	100	12	CT26	In vitro and in vivo	√	√	√	√	-	[74]
26.		siRNA, carboxylate grapheme oxide, trimethyl C, HA	95	27.2	CT26	In vitro	√	√	√	-	-	[75]
27.	Oral squamous cancer	Paclitaxel, PCL, C, HA	257	−25	EC109	In vitro and in vivo	√	-	√	√	√	[76]
28.		Cathecol, C, HA	160	−12.7	HN22	In vitro	√	√	√	-	-	[77]
29.	Bladder cancer	siRNA, C, HA	100–120	30–40	T24	In vitro	√	-	√	-	-	[23]
30.		siRNA, Dox, TAT peptide, C, HA	118	9	T24	In vitro and in vivo	√	-	√	√	-	[78]
31.		siRNA and the EP4 antagonist, C, HA	130	27	T24	In vitro	√	-	√	-	-	[79]
32.	Others	Irinotecan, 5-fluorouracil, PLGA, C, HA	153	−13.7	MGC803	In vitro and in vivo	√	-	√	√	-	[80]
33.		Nitric oxide, C, HA	170	15	PC-3	In vitro	√	-	-	-	-	[81]
34.		Graphene oxide, fluorescein isothiocyanate, C, HA	200	−41	HeLa	In vitro	√	-	√	-	-	[82]
35.		Curcuminoid, C, HA	210–240	25	C6	In vitro	√	-	√	-	-	[83]
36.		Dox, nitric oxide, C, HA	170–200	−39–(−47)	HeLa	In vitro	√	-	√	-	-	[84]

Abbreviations: NDDS: nanoparticle drug delivery system; Rs: In vitro release; ITG: inhibition tumor growth; Ap: induce apoptosis; CU: increase cellular uptake; TV: decrease tumor volume; S: increase selectivity; Dox: doxorubicin; DTX: docetaxel; PCL: polycaprolactone; PLGA: poly lactic co glycolic acid; C: chitosan; HA: hyaluronic acid.
